# Serial platelet level index improves prediction of pulmonary hemorrhage in patients with *Stenotrophomonas maltophilia* respiratory infections and thrombocytopenia

**DOI:** 10.3389/fmed.2022.940159

**Published:** 2022-09-02

**Authors:** Huai-Chueh Gem Wu, Huai-Shing Wu, Chao-Neng Cheng, Jiann-Shiuh Chen, Tsai-Yun Chen, Chung-I Li, Ching-Fen Shen

**Affiliations:** ^1^Department of Internal Medicine, Chang Gung Memorial Hospital, Taoyuan, Taiwan; ^2^Education Center, National Cheng Kung University Hospital, Tainan, Taiwan; ^3^Department of Computer Science and Information Engineering, National Taiwan University, Taipei, Taiwan; ^4^Department of Pediatric, National Cheng Kung University Hospital, College of Medicine, National Cheng Kung University, Tainan, Taiwan; ^5^Department of Internal Medicine, National Cheng Kung University Hospital, College of Medicine, National Cheng Kung University, Tainan, Taiwan; ^6^Department of Statistics, College of Management, National Cheng Kung University, Tainan, Taiwan

**Keywords:** *Stenotrophomonas maltophilia* respiratory infection, hemorrhagic pneumonia, pulmonary hemorrhage, platelet d-index, thrombocytopenia

## Abstract

Patients with thrombocytopenia (platelet count <150 × 10^3^/μL) often develop pulmonary hemorrhage (PH) after *Stenotrophomonas maltophilia* (SM) respiratory infection, resulting in a high respiratory failure rate and increased mortality. Developing an efficient method for early prediction of PH in these patients may improve survival. This study aimed to evaluate risk factors in PH and to develop an index measuring serial platelet deficit to predict PH in patients with SM respiratory infection. Data of patients with SM respiratory infection and thrombocytopenia treated in a tertiary university hospital during 2018–2020 were retrospectively retrieved from electronic medical records and analyzed. SM respiratory infection was defined as SM isolated from sputum, endotracheal suction, or bronchial alveolar lavage plus acute respiratory symptoms. Between PH and non-PH groups, clinical characteristics and laboratory parameters were collected and compared. The newly developed platelet dissimilarity index (d-index) was calculated by accumulating differences between the actual and the lowest normal level of the platelet count in each patient at different time points. Within 1,039 patients with positive SM culture, 437 cases matched the criteria and were analyzed. A total of 125 (28.6%) patients developed PH and 312 (71.4%) did not. The patients with PH had increased prothrombin time/international normalized ratio (PT/INR), lower platelet count, and higher platelet d-index. Multivariate analysis revealed that extreme thrombocytopenia (platelet count <50 × 10^3^/μL) is a common independent risk factor in PH and mortality. The performance of platelet deficit and d-index varied between patients with different comorbidities. Performance of platelet deficit to predict PH is better in patients with hematology/oncology or liver disease (area under curve, 0.705–0.757), while d-index is better in patients with sepsis/treatment and various other groups (0.711–0.816). Prolonged and extreme thrombocytopenia is a determinant risk factor in PH in patients with SM respiratory infection. Given the complexity of causes of thrombocytopenia and associated comorbidities, different strategies should be applied when assessing the risk for PH.

## Introduction

*Stenotrophomonas maltophilia* (SM) is a ubiquitous, globally emerging, multiple-drug resistant, non-fermenting gram-negative bacillus (NFGNB) that typically causes opportunistic infection in severely immunocompromised or debilitated patients. Respiratory infection, including ventilator-associated pneumonia (VAP), is the most common clinical presentation and potentially results in pulmonary hemorrhage (PH) (1). The extracellular protease secreted by SM is reported to cause tissue destruction, vascular damage, and subsequent hemorrhage (2–4). Although PH is an uncommon complication of SM respiratory infection, previous studies have reported mortality as high as 85% within 30 days of the infection onset (5–10).

Risk factors in PH have been identified in different clinical settings and patients with various comorbidities. Hematologic malignancy and thrombocytopenia were found to be predictive factors of hemorrhagic pneumonia in patients with SM bacteremia (5, 11). Also, neutropenia, longer duration of neutropenia, high C-reactive protein (CRP) or procalcitonin (PCT) levels, and persistent fever despite using broad-spectrum antibiotics are independent predictors of PH in patients who received hemopoietic cell transplantation (HCT) or other hematologic diseases (7, 8). Although thrombocytopenia has been identified as an important risk factor in hemorrhagic complications in general practice, no comprehensive evaluation has yet addressed the degree of thrombocytopenia and its association with PH in SM infections. The cumulative d-index (c-d-index) is a scoring system that uses absolute neutrophil counts over the course of neutropenia to characterize the degree and durability of neutropenia; the index has been applied to febrile neutropenic patients to assess the risk of invasive mold infections (12–14). The present study aimed to develop an index of serial platelet levels from different time windows to evaluate the degree of thrombocytopenia and explore its associations with PH in respiratory SM infections.

## Methods

### Patients

The electronic medical records from National Cheng Kung University Hospital, Tainan, Taiwan, were searched using the criteria of hospitalized patients with SM isolated from sputum, endotracheal suction, or bronchial alveolar lavage culture between January 2018 and December 2020. SM respiratory infection was defined as positive culture, plus newly respiratory symptoms, pulmonary infiltrates on chest X-ray, or increased oxygen demand. Only data of patients with SM respiratory infection and thrombocytopenia (platelet count <150 × 10^3^/μL) 7 days before and after the date of sputum culture collection were extracted from the medical records database. The patients with duplicate culture results within the same hospitalization period were merged as a single event. Cases with (1) a normal or high platelet count 7 days before and after the date of sputum culture collection, (2) evidence of pulmonary invasive fungal infection, (3) only Stenotrophomonas colonization without respiratory symptoms, or (4) incomplete clinical data, were excluded. The Institutional Review Board of National Cheng Kung University Hospital reviewed and approved the study protocol (IRB No. A-ER-110-079). Owing to the study's retrospective nature, signed informed consent from the patients was waived.

### Clinical variables and data collection

Demographic and clinical data, including age, sex, reason for admission, existence of hematological disease, alternation or disruptions in pulmonary structure (e.g., lung cancer, intrathoracic surgery, tuberculoma), existence of neutropenia (absolute neutrophil count <500/μl), PT/APTT ratio within a week (APTT/MNAPTT >1.2 and INR >1.2 were considered prolonged), the platelet count on the day of sputum culture, and use of mechanical ventilation, were extracted from the electronic medical records. Causes of thrombocytopenia were grouped as follows: sepsis-medication related (“Sepsis-Rx”) (e.g., disseminated intravascular coagulation due to severe infection, medication-related platelet destruction); hematological disease and chemotherapeutics use due to oncological disease (“Hem-Onc”); liver disease (“Liver”); and various other causes (“Others”) (e.g., mechanical destruction, transfusion consumption coagulopathy or major bleeding, unknown cause). PH was defined in the patients with multiple episodes of hemoptysis and desaturation or evidence of deteriorating pulmonary function or anatomy (e.g., increased infiltrations in chest X-ray, increased oxygen demand).

### Platelet d-index and platelet deficit calculation

d-index was applied in patients with prolonged neutropenia and opportunistic infections using an accumulated difference between the neutrophil count and the threshold of neutropenia, as described previously (12–14). The same rationale was applied to estimate the accumulated platelet deficit in thrombocytopenic patients. The platelet d-index was calculated using this algorithm.


(1) $$Platelet\;D_{index}=\;A_{e}-\;A_{0}=\;\sum_{i=2}^{n}\left[150*\left(t_{i}-t_{i-1}\right)\right]-\sum_{i=2}^{n}\left[\frac{P_{i-1}+P_{i}}{2}*(t_{i}-t_{i-1})\right]$$

Accumulated platelet deficit between estimated area under curve (Ae) and observed area under curve (Ao) 7 days before and after sputum culture result of SM was calculated. Time window of platelet d-index included 3 days before and after the SM sputum test date (d-index-6), 5 days before and after (d-index-10), and 7 days before and after (d-index-14). The platelet deficit was calculated using the lowest level of the normal platelet count (150 x 10^3^/μl) minus the platelet count at the test date. These d-index values were divided by number of the days on which data were available so that comparison between different time intervals could be available.

### Statistical analysis

All statistical analyses were performed using SPSS 17 for Windows (IBM SPSS, Armonk, NY, USA). The patients with SM respiratory infections were grouped as “with PH” or “without PH.” The independent sample *t*-test was used for continuous variables, and the Chi-square test or the Fisher's exact test was used for dichotomous variables. *P*-value < 0.05 was considered statistically significant. Cox's proportional hazard analysis was used to determine risk factors in PH and 30-day mortality. One-way ANOVA was used to assess platelet levels and d-index of various causes of thrombocytopenia. To assess the ability of platelet d-index for different time windows and platelet deficits of a single day to predict pulmonary hemorrhage, sensitivity and specificity were analyzed using receiver operating characteristic (ROC) curves across the sputum sampling day, showing SM positive. The optimal cut-off value was determined by the maximum sums of sensitivity and specificity.

## Results

### Study population

**Figure 1:**
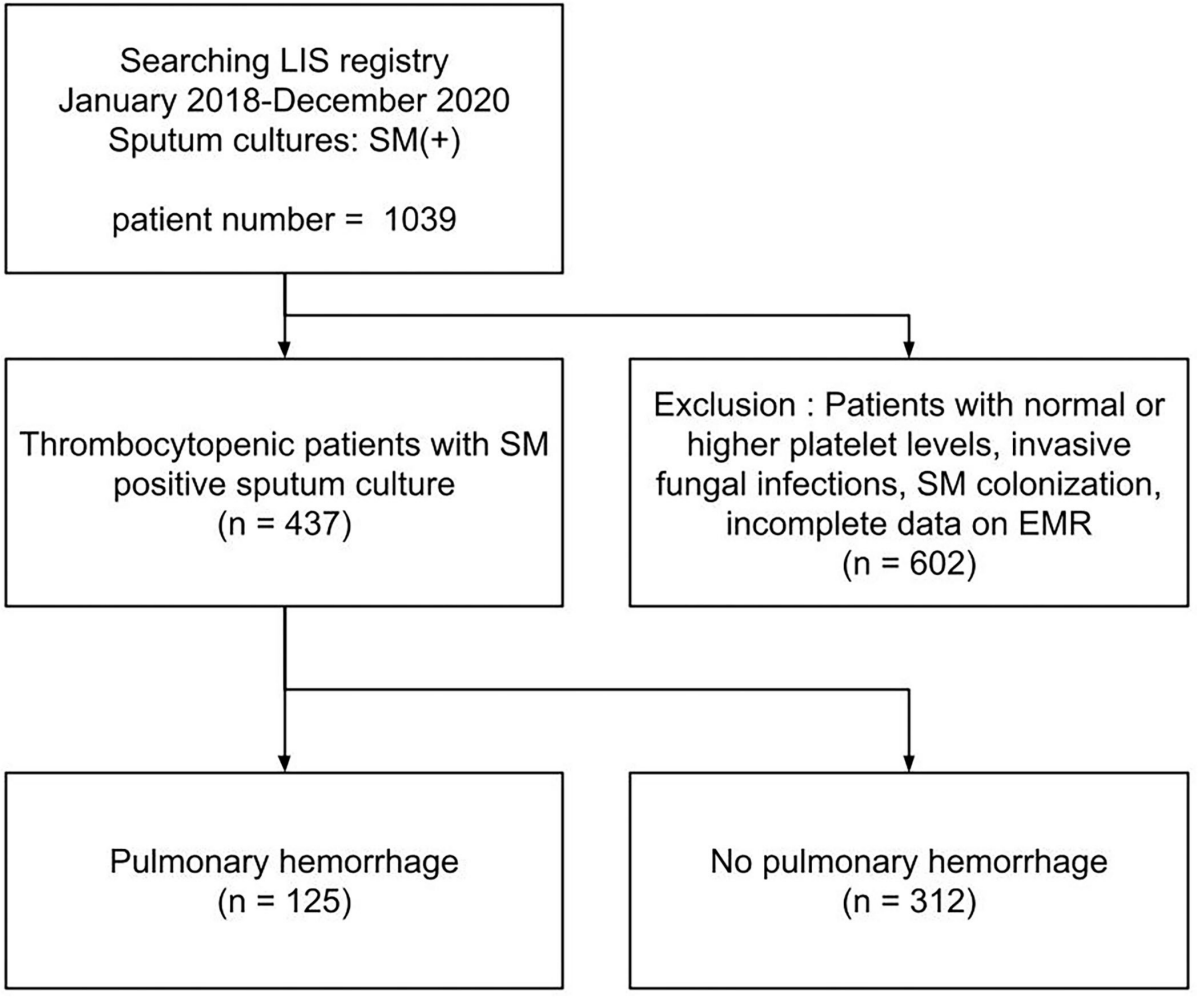
A flow chart of study population. LIS, laboratory information system; EMR, electronic medical record system.

Of the 437 patients with thrombocytopenic SM respiratory infection, 125 patients (28.6%) developed PH (shown in Figure 1). The patients' demographic and clinical characteristics are shown in Table 1. No significant differences were found in age, sex, and cause of thrombocytopenia between the patients with and without PH. The most common causes of thrombocytopenia were “Sepsis-Rx” (57%), followed by “Others” (15.1%), “Liver disease” (14.9%), and “Hem-Onc” (13.%). Although hematological disease accounts for the majority (48/57, 84.2%) of cases in the “Hem-Onc” group, the proportion of hematological diseases was not significantly different between the “Hem-Onc and “Liver” groups. Platelet counts at the test date were lower, and the d-index of different time frames was higher in the PH group than in the non-PH group (*p* < 0.001). Also, prolonged PT/INR and mechanical ventilation use were significantly associated with PH (all *p* < 0.05). The mortality of SM respiratory infection was higher in the PH group than in the non-PH group (87.2 vs. 57.7%, *p* < 0.001), with the overall rate of 66.1%. Half of the patients with PH died within the 1st week after diagnosis, and the mortality rate declined in the 2nd and 3rd weeks. None of the neutropenic patients with PH (*n* = 15) survived.

**Table 1:** Demographic and clinical characteristics of patients with SM respiratory infections.

**Characteristics**	**All patients (*N* = 437)**	**Patients without PH (*n* = 312)**	**Patients with PH (*n* = 125)**	***p*-value**
Male, no. (%)	273 (62.5)	187 (59.9)	86 (68.8)	0.084
Age, years, median (IQR)	69 (59–79)	69 (59–80)	70 (59–78)	0.467
**Comorbidities associated with thrombocytopenia, no. (%)**				0.377
Hema-Onc	57 (13.0)	35 (11.2)	22 (17.6)	
Liver disease	65 (14.9)	46 (14.7)	19 (15.2)	
Sepsis-Rx	249 (57.0)	185 (59.3)	64 (51.2)	
Others	66 (15.1)	46 (14.7)	20 (16.0)	
Pulmonary structural abnormalities, no. (%)	160 (36.6)	112 (35.9)	48 (38.4)	0.624
Mechanical ventilation, no. (%)	362 (82.8)	250 (80.1)	112 (89.6)	**0.018**
Neutropenia, no. (%)	43 (9.8)	28 (9.0)	15 (12.0)	0.337
APTT/MNAPTT ratio, median (IQR)	1.04 (0.93–1.25)	1.01 (0.91–1.18)	1.14 (0.99–1.34)	0.506
PT-INR, median (IQR)	1.31 (1.16–1.57)	1.28 (1.14–1.50)	1.36 (1.22–1.69)	0.007
Platelet count at test date, mean (range) (10^3^/**μ**L)	85.17 (6–399)	92.30 (6–324)	67.90 (6–399)	**<0.001**
**d-index per day, mean**				
d-index-14	68.49	60.22	88.98	**<0.001**
d-index-10	68.75	60.18	90.31	**<0.001**
d-index-6	70.23	61.67	91.78	**<0.001**
**Mortality, no. (%)**	289 (66.1)	180 (57.7)	109 (87.2)	**<0.001**
within 7 days	132 (30.2)	70 (22.4)	62 (49.6)	**<0.001**
within 14 days	190 (43.5)	102 (32.7)	88 (70.4)	**<0.001**
within 30 days	229 (52.4)	130 (41.7)	99 (79.2)	**<0.001**

Data are shown as no. (%), median (IQR), and mean (range).

Hema-Onc, thrombocytopenia due to hematology/oncology diseases or the associated chemotherapeutics; liver disease, thrombocytopenia due to liver diseases; sepsis-Rx, thrombocytopenia due to sepsis or medications; others, thrombocytopenia due to causes other than those described above; Pulmonary structural abnormalities indicated patients with alternation or disruptions in pulmonary structure (e.g., lung cancer, intrathoracic surgery, tuberculoma); neutropenia, absolute neutrophil count <500/ul; APTT/MNAPTT, activated partial thromboplastin time/mean normal-activated partial thromboplastin time; PT/INR, prothrombin time and international-normalized ratio; d-index-14, d-index 7 days before and after the sputum test date; d-index-10, d-index 5 days before and after the sputum test date; d-index-6, d-index 3 days before and after the sputum test date. The *p*-value < 0.05 was marked in bold.

### Risk factors associated with pulmonary hemorrhage and mortality

Table 2 and Supplementary Table 2 list the results of Cox proportional hazard analysis of risk factors associated with PH in neutropenic SM respiratory infections and risk factors associated with 30-day mortality in the PH group, respectively. In univariate analysis, neutropenia and extreme thrombocytopenia (the platelet count at the test date, <50 × 10^3^/μl) were both risk factors in PH and 30-day mortality. However, multivariate analysis showed that severe thrombocytopenia was the only independent risk factor in both PH and 30-day mortality in patients with PH.

**Table 2:** Univariate and multivariate analysis of risk factors associated with pulmonary hemorrhage in patients with SM respiratory infections.

**Characteristics**	**Univariate analysis**		**Multivariate analysis**	
	**HR (95% CI)**	***p*-value**	**aHR (95% CI)**	***p*-value**
Age >65 years	1.032 (0.790–1.621)	0.499		
Male sex	1.176 (0.805–1.718)	0.402		
Neutropenia	2.005 (1.145–3.511)	**0.015**	1.399 (0.725–2.699)	0.316
Pulmonary structural abnormalities	1.013 (0.705–1.456)	0.943		
Mechanical ventilation	0.858 (0.480–1.533)	0.605		
APTT/MNAPTT > 1.2	1.080 (0.720–1.621)	0.709	0.971 (0.615–1.532)	0.899
INR > 1.2	1.066 (0.661–1.721)	0.793	1.062 (0.590–1.912)	0.842
Platelet at test date <50 x10^3^/μL	1.787 (1.230–2.596)	**0.002**	2.310 (1.346–3.965)	**0.002**

Neutropenia, absolute neutrophil count <500/ul. Pulmonary structural abnormalities indicated patients with alternation or disruptions in pulmonary structure (e.g., lung cancer, intrathoracic surgery, tuberculoma). APTT/MNAPTT, activated partial thromboplastin time/mean normal-activated partial thromboplastin time; PT/INR, prothrombin time and international normalized ratio. HR, hazard ratio; aHR, adjusted hazard ratio; 95% CI (confidence interval). The *p*-value < 0.05 was marked in bold.

### Characteristics of SM respiratory infections by cause of thrombocytopenia

Comparison of clinical characteristics and laboratory findings between patients with thrombocytopenic SM respiratory infection caused by different comorbidities is shown in Table 3. The “Sepsis-Rx” group had more patients aged 65 years and older (67.9%), higher pulmonary structural abnormalities (41.8%), and greater need for mechanical ventilation (86.7%). Platelet counts at the test date were significantly lower in patients in “Hem-Onc” and “Liver” groups than those in the “Sepsis-Rx” and “Others” groups, with the most extreme thrombocytopenia (platelets, <50 × 10^3^/μl) cases in the “Hem-Onc” group. The d-index-14 was inversely correlated with platelet counts at the test date and was significantly higher in patients in the “Hem-Onc” and “Liver” groups than those in the “Sepsis-Rx” and “Others” groups. However, the percentage of PH and the time to developing PH from diagnosis were not significantly different between these four groups. Mortality rates (66.1%) were significantly higher in the “Hem-Onc” (71.9%) and “Sepsis-Rx” (71.9%) groups than those in the “Liver” (52.3%) and “Others” groups (53%).

**Table 3:** Comparison of clinical characteristics and laboratory findings between patients with SM respiratory infections by different comorbidities.

	**Overall** **(*N* = 437)**	**Hema-Onc (*n* = 57)**	**Liver** **(*n* = 65)**	**Sepsis-Rx (*n* = 249)**	**Others** **(*n* = 66)**	***p*-value**
Age > 65-year-old, no. (%)	264 (60.4)	28 (49.1)	29 (44.6)	169 (67.9)	38 (57.6)	**0.001**
Male sex, no. (%)	273 (62.5)	34 (59.6)	42 (64.6)	156 (62.7)	41 (62.1)	0.955
Pulmonary structural abnormalities, no. (%)	160 (36.6)	15 (26.3)	13 (20)	104 (41.8)	28 (42.4)	0.003
Mechanical ventilation, no. (%)	362 (82.8)	37 (64.9)	49 (75.4)	216 (86.7)	60 (90.9)	**<0.001**
APTT/MNAPTT > 1.2, no. (%)	92 (28.9)	11 (26.8)	20 (40)	47 (26.6)	14 (28.0)	0.311
INR > 1.2, no. (%)	222 (66.3)	31 (72.1)	46 (79.3)	116 (62.7)	29 (59.2)	0.065
Platelet count at test date (10^3^/μL), mean (95%CI)	85.3 (80.3–90.3)	45.82 (35.0–56.7)	65.62 (56.6–74.7)	94.93 (88.5–101.4)	102.59 (88.1–117.1)	**<0.001**
Percentage of extreme thrombocytopenia ( ≤ 50 x10^3^/μL), no. (%)	9 (2.1)	7 (12.3)	0 (0)	1 (0.4)	1 (1.5)	**<0.001**
d-index-14, mean (95%CI)	68.33 (64.4–72.2)	104.08 (94.6–113.5)	83.04 (75.8–90.9)	59.79 (54.7–64.9)	55.21 (45.77–64.7)	**0.008**
Pulmonary hemorrhage, no. (%)	125 (28.6)	22 (38.6)	19 (29.2)	64 (25.7)	20 (30.3)	0.270
Time to development of PH, mean days (95%CI)	5.9 (3.6–8.3)	3.0 (0.4–5.6)	3.0 (1.2–4.8)	7.8 (3.8–11.9)	6.0(0.0–12.3)	0.148
Death, no. (%)	289 (66.1)	41 (71.9)	34 (52.3)	179 (71.9)	35 (53)	**0.002**

Data are shown as no. (%) or mean (95% CI, confidence interval). Hema-Onc, thrombocytopenia due to hematological diseases or the associated chemotherapeutics; liver, thrombocytopenia due to liver diseases; sepsis-Rx, thrombocytopenia due to sepsis or medications; others, thrombocytopenia due to other causes than the described above. Pulmonary abnormalities indicated patients with alternation or disruptions in pulmonary structure (e.g., lung cancer, intrathoracic surgery, tuberculoma). APTT/MNAPTT, activated partial thromboplastin time/mean normal-activated partial thromboplastin time; PT/INR, prothrombin time and international normalized ratio. (Available data of APTT n = 318, PT n = 335.) d-index-14, d-index 7 days before and after the sputum test date. The *p*-value < 0.05 was marked in bold.

### Platelet count-derived clinical parameters in predicting PH

**Figure 2:**
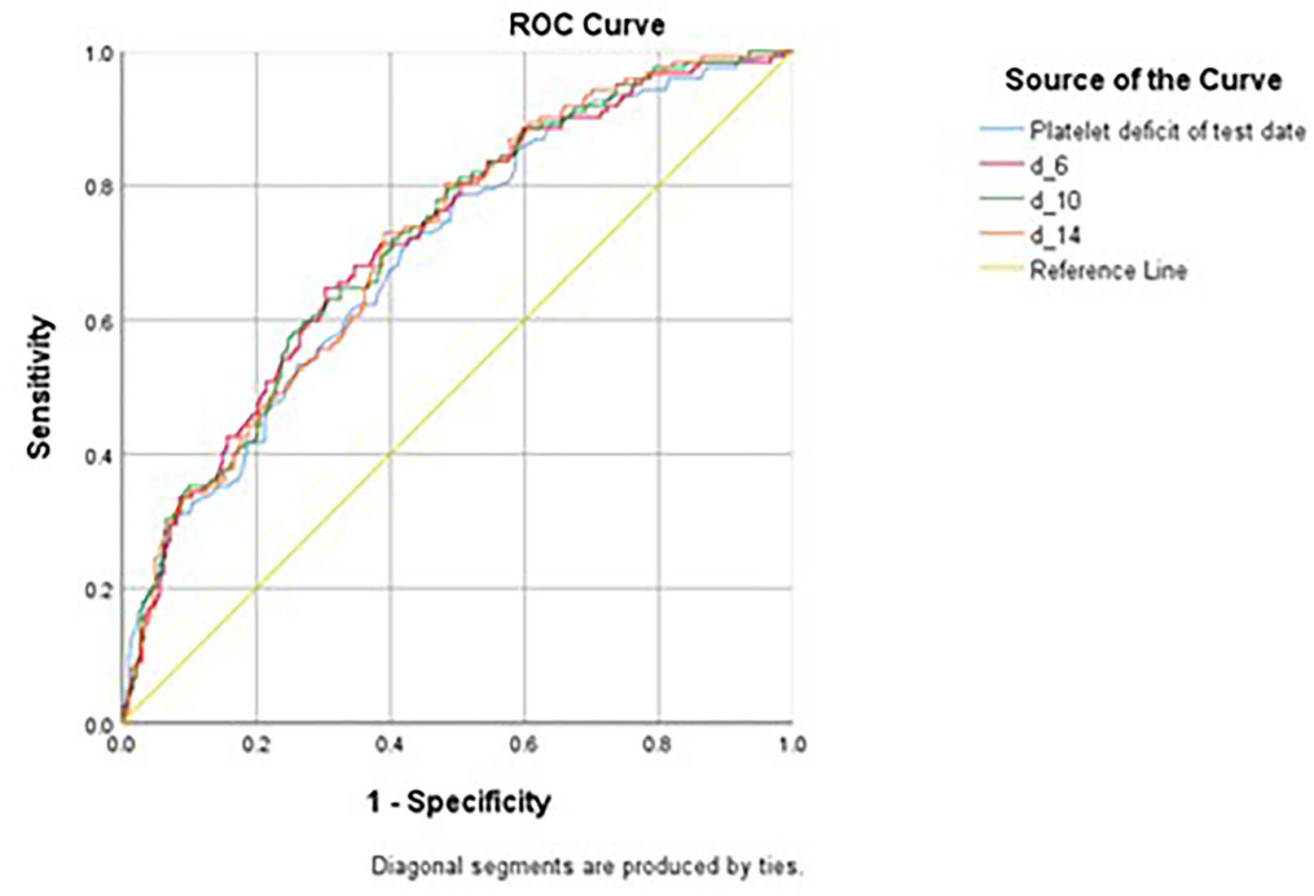
Receiver Operating Curve (ROC) of various measurements in predicting pulmonary hemorrhage in patients with SM infection. Platelet deficit of the test date, the deficit of the platelet count from the lowest normal limit on the test date (150 × 10^3^/μL - platelet count). d_6, daily average of d-index 3 days before and after the sputum test date, d_10, daily average of d-index 5 days before and after the sputum test date, d_14, daily average of d-index 7 days before and after the sputum test date.

Various platelet count-derived clinical parameters were used to predict PH in patients with thrombocytopenia SM respiratory infection, and the performance is shown in Figure 2 and Table 4. The predictability of these parameters varied across different causes of thrombocytopenia, and d-index-10 had the highest AUC (0.719) in all cases. Platelet deficit performed better than d-index in the “Hem-Onc” and “Liver” groups, and the two groups combined. In contrast, d-index performed better in the “Sepsis-Rx” and “Others” groups. D-index-14 of the “Others” group showed the highest rate of predictability (AUC = 0.816) of PH across all groups. The cut-off values of platelet deficit and d-index-14 according to the maximum sums of sensitivity and specificity are listed in Table 4. The optimal cut-off value of platelet deficit on the test date for all cases was 70.5 × 10^3^/μl, corresponding to platelet levels of 79.5 × 10^3^/μl, with a sensitivity of 69.8% and a specificity of 57.7% in predicting PH. The optimal platelet cut-off value at the test date varied across the four groups, from 17.5 × 10^3^/μl in the “Hem-Onc” group to 67.5 × 10^3^/μl in the “Liver” group, 86.5 × 10^3^/μl in the “Sepsis-Rx” group, and 106 x 10^3^/μl in the “Others” group. We subtracted the d-index-14 by days to calculate the average platelet deficit per day, and the corresponding value had a similar pattern as the platelet deficit at the test date, but with more reliability in “Sepsis-Rx” and “Others” group.

**Table 4:** Performance of various measurements in predicting pulmonary hemorrhage in patients with SM respiratory infection by different comorbidities.

**AUC**	**All**	**Hema-Onc & liver**	**Sepsis-Rx & others**	**Hema-Onc**	**Liver**	**Sepsis-Rx**	**Others**
Platelet deficit of test date	0.699	**0.729**	0.684	**0.757**	**0.705**	0.664	0.757
d-index-6	0.717	0.691	0.724	0.676	0.689	0.701	0.808
d-index-10	**0.719**	0.688	**0.734**	0.698	0.674	**0.712**	0.807
d-index-14	0.714	0.672	**0.734**	0.688	0.648	0.711	**0.816**
**Platelet deficit at test date**							
Cut-off value, 10^3^/uL	70.5	118.5	48.5	132.5	82.5	63.5	44.0
Sensitivity	69.8%	53.7%	77.4%	50.0%	84.2%	68.8%	85.0%
Specificity	57.7%	81.5%	48.9%	88.6%	52.2%	59.5%	54.3%
**Platelet deficit derived from d-index-14**							
Cut-off value, 10^3^/uL	71.3	117.3	71.3	117.3	92.4	71.3	71.1
Sensitivity	70.6%	46.3%	63.1%	63.6%	63.2%	60.9%	70.0%
Specificity	61.5%	87.7%	73.4%	77.1%	67.4%	73.5%	73.9%

AUC, area under curve; cut-off value(/μL) indicates the platelet level in specific group that could reach the optimal sum of highest sensitivity and specificity; platelet deficit on the test date indicated the deficit of the platelet count from the lowest normal limit (platelet count, 150 x 10^3^/uL) on the test date. The *p*-value < 0.05 was marked in bold.

## Discussion

Although thrombocytopenia had been recognized as a significant risk factor in PH in SM respiratory infection, in the present study, the degree of thrombocytopenia by platelet deficit at a single time point and over a certain period was evaluated to identify the optimal cut-off value in predicting PH. Also, results of the present study demonstrated the clinical heterogeneity of thrombocytopenia as the cause and its association with PH, which may explain the conflicting findings of previous observational studies and address the need for more precise and individualized risk classification based on comorbidities. Different measurement tools should be used in distinct subpopulations to predict PH; for example, platelet counts at the test date should be sufficient in patients with hematological and liver diseases, while d-index using serial platelet measurements performs more reliably for those with sepsis and other causes.

Mortality associated with PH was overwhelmingly high in the present study, especially in patients with hematologic malignancy. Recently, increasing numbers of cases have been successfully treated, which may be due to refinement of preventive measures of transmission, rising awareness of SM infections, and timely use of empirical antimicrobial therapy (15, 16). The mortality rate in the present study was highest within the 1st week of diagnosis (50%), demonstrating the potential fulminant behavior of SM infections. Notably, only one patient with hematological disease survived through hemorrhagic SM respiratory infection, and none of the neutropenic patients survived. In a previous study conducted by Bao et al. (17) patients with severe prolonged neutropenia and thrombopenia due to hematological disorders were shown to have 100% mortality when encountering SM bacteremia. The present study echoes their findings, indicating that hematological abnormalities complicate the clinical course of SM respiratory infection and worsen the disease outcome. Although neutrophil counts may have been a decisive factor in infection control and hematopoiesis ability, it was also shown to be a significant risk factor in PH in previous studies (18). After multivariate analysis, we found that neutropenia was not an independent risk factor in PH or associated mortality, implying that neutropenia is, probably, a collateral finding along with underlying disease and thrombocytopenia.

The patients in the “Sepsis-Rx” and “Others” groups with SM-associated PH had higher proportions of pulmonary structural abnormalities and mechanical use. Because these patients are likely to receive mechanical ventilation due to pulmonary structure anomaly, they acquired SM infection through ventilator use. Until now, the attributable factors causing SM-related VAP have remained unclear (1, 18). A recent study has found that exposure to ureido/carboxypenicillin or carbapenem during the week before VAP and the severity of disease leading to respiratory and hematological failures were independent risk factors in SM-VAP (1). Moreover, SM-VAP mortality remains high even in patients receiving adequate treatment, either monotherapy or combinations of antimicrobials.

Platelet counts at a single time point or over a given time period were significantly lower in patients with hematological and liver diseases than in those with sepsis or other diseases, but risk for PH did not differ significantly between these patients. Although thrombocytopenia is an independent determining factor in PH, patients experiencing long-term thrombocytopenia may develop a certain mechanism by which to compensate for the bleeding tendency. A previous study has identified that an increased number of larger-sized platelets may compensate for the impaired platelet function in patients with chronic idiopathic thrombocytopenia (19). In patients with acute myeloid leukemia who have thrombocytopenia, platelet aggregation, and platelet activation predicted bleeding better than the platelet count alone (15). Overall, the bleeding risk is not only dependent on the platelet count but also on the platelet function, coagulopathy, and the underlying disease causing thrombocytopenia. Further mechanistic exploration of the cause of reduced platelet counts or function in different thrombocytopenic conditions is still needed to help develop and implement preventive strategies (16).

In the present study, thrombocytopenia due to hematological and liver diseases both had prolonged and profound low platelet levels and PH usually developed at the nadir of the platelet level. Meanwhile, the pattern of thrombocytopenia due to sepsis/medication or other causes was more alike and had a higher average platelet count compared to those in hematological and liver disease. Traditionally, the pathophysiology of thrombocytopenia in chronic liver disease has long been attributed to hypersplenism, where pooling and sequestration of blood result in platelet consumption. Recently, other mechanisms, including bone marrow suppression by toxic substances, such as alcohol or viral infection, may also contribute to thrombocytopenia. In addition, the thrombopoietin, predominantly produced by the liver, is markedly reduced in advanced-staged liver disease, which also contributes to reduced thrombopoiesis in the bone marrow (20). All mechanisms mentioned above can explain the thrombocytopenia caused by liver disease that may be secondary to bone marrow suppression, similar to those in hematological disorders.

The clinical consensus regarding the lowest platelet level needed to prevent bleeding in certain circumstances is that platelet counts must be above 20–50 × 10^3^/μl for bronchoscopy exams and 50 × 10^3^/μl for transbronchial lung biopsies (21). Statistical results from the present study show that platelet levels above 60–100 × 10^3^/μl may plausibly prevent PH in patients with sepsis, liver disease, and other causes, while lower platelet counts (above 17.5 × 10^3^/μl) may be tolerated in patients with hematological diseases. In addition to certain cut-off levels for platelet counts, the accumulative platelet deficit over a given time period was found to be more reliable in predicting PH in patients with sepsis, medication related, or other causes of thrombocytopenia. Although these groups of patients did not have thrombocytopenia as profound as in those with hematological disorders, they are more susceptible to developing PH in prolonged thrombocytopenia. Therefore, regular prophylactic platelet transfusion to maintain platelet levels above certain thresholds over unstable periods may be a potential strategy to prevent PH in this group of patients.

The present study used the largest database, evaluating PH in patients with SM respiratory infections, and is also the first study to extrapolate serial platelet measurements in predicting PH. Data in the present study provide clinicians with the optimal cutoff platelet level for transfusion therapy to prevent PH in thrombocytopenia of various causes. The concept of using d-index as a personalized measurement in evaluating the severity of thrombocytopenia can be implemented to prevent SM hemorrhagic pneumonia and bleeding disorders in various other diseases. Especially in the era of the coronavirus disease 2019 (COVID-19) pandemic, clinicians may also utilize the d-index to assess the risk of PH in sepsis due to COVID-19.

Regardless of the above strengths, the present study has several limitations. First is that we did not evaluate associations between PH, antibiotic use, and corresponding antimicrobial susceptibility. Although in both clinical and animal studies, timely appropriate antibiotic use could reduce the risk of mortality in SM hemorrhagic pneumonia, systematic review demonstrated that even patients treated with appropriate antibiotics, including trimethoprim/sulfamethoxazole, fluoroquinolones, or both combined, the mortality remained high, even reaching 100% (11, 22). Also, no prior antimicrobial therapy for SM bacteremia had shown a preventive effect for PH (7). Still, prompt administration of antibiotics is essential for control of infectious disease, but the use of antibiotics in preventing PH in SM respiratory infection does not appear to be an adequate measure. Secondly, the mean platelet volume (MPV) level was not included in our study. Large MPV had reflected poor prognosis in various diseases, increased risk of hemorrhage and thromboembolism. Further investigation might be needed to elucidate the MPV, as well as platelet function with PH. Thirdly, total number of patients with positive blood culture of SM was unavailable to attain, which was also a limitation of our study. SM bacteremia are mostly resulting from indwelling catheters (73%), and only 22% are secondary to other causes (23). However, identification of bacteremia in SM respiratory infection would help to evaluate disease severity and strengthen diagnosis.

## Conclusion

Prolonged and extreme thrombocytopenia is a determinant risk factor in PH in patients with SM respiratory infections. The degree of platelet deficit varies significantly between different causes of thrombocytopenia and types of underlying comorbidities. Single time-point measurement of platelet counts may most reliably predict PH in patients with hematological and liver diseases, while serial measurement over a given time period may reliably predict PH in those with sepsis and other causes of thrombocytopenia.

## Data availability statement

The raw data supporting the conclusions of this article will be made available by the authors, without undue reservation.

## Author contributions

H-CW and C-FS conceived the original study. C-FS oversaw data collection, oversaw the analysis, writings, and figures. H-CW acquired all data, analyzed the data, wrote the original manuscript, and prepared the final figures. H-SW wrote the d-index algorithm. Statistical analysis was overseen by C-IL and C-FS. C-NC, J-SC, and T-YC provided some insights into clinical association. All the authors have seen and approved the final version of the manuscript.

## Funding

This work was supported by grants from the Clinical Medical Research Center, National Cheng Kung University Hospital, Taiwan (NCKUH-11102024), and Ministry of Science and Technology, Taiwan (110-2923-B-006-001-MY4).

## Conflict of interest

The authors declare that the research was conducted in the absence of any commercial or financial relationships that could be construed as a potential conflict of interest.

## Publisher's note

All claims expressed in this article are solely those of the authors and do not necessarily represent those of their affiliated organizations, or those of the publisher, the editors and the reviewers. Any product that may be evaluated in this article, or claim that may be made by its manufacturer, is not guaranteed or endorsed by the publisher.
