# Supplementary figures and images for: Serial platelet level index improves prediction of pulmonary hemorrhage in patients with Stenotrophomonas maltophilia respiratory infections and thrombocytopenia
Source: Front Med (Lausanne). 2022 Sep 2;9:940159. doi: 10.3389/fmed.2022.940159 (PMC9486816; doi:10.3389/fmed.2022.940159)

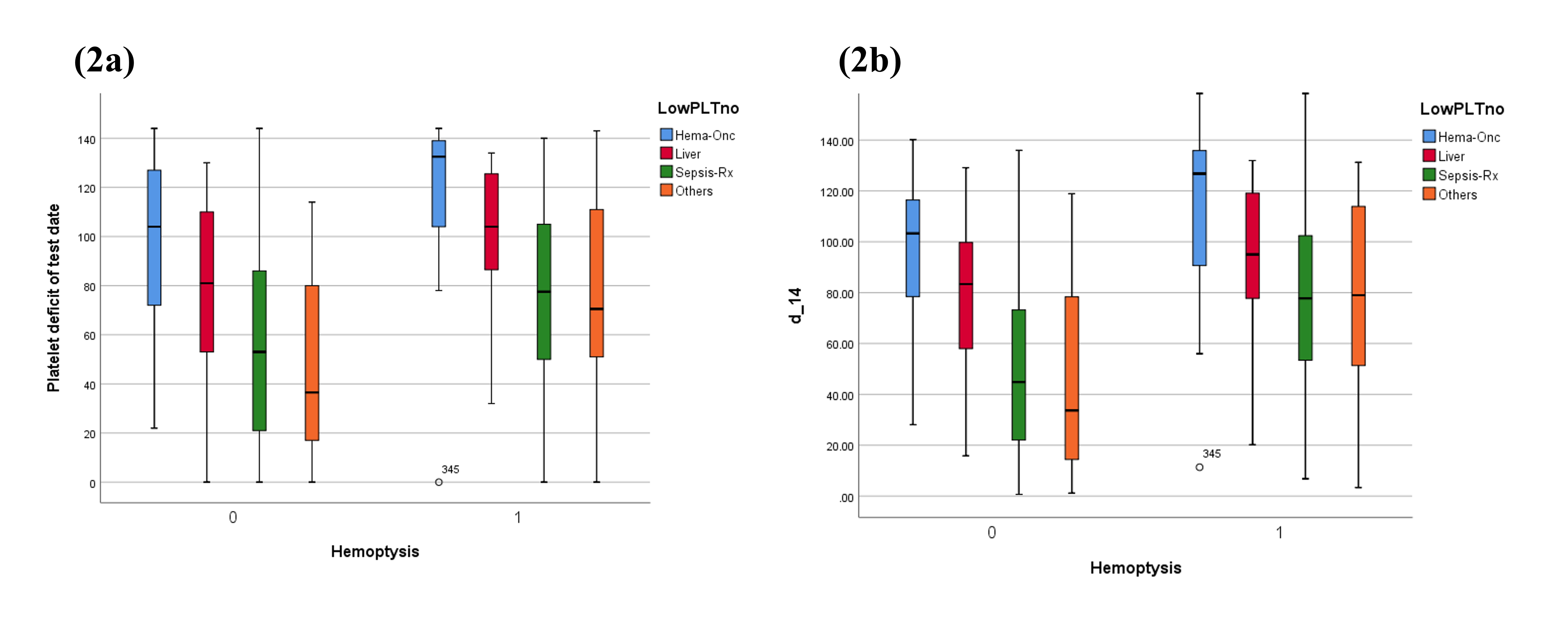

Supplement: Supplementary file 3 [file Image_1.PNG]

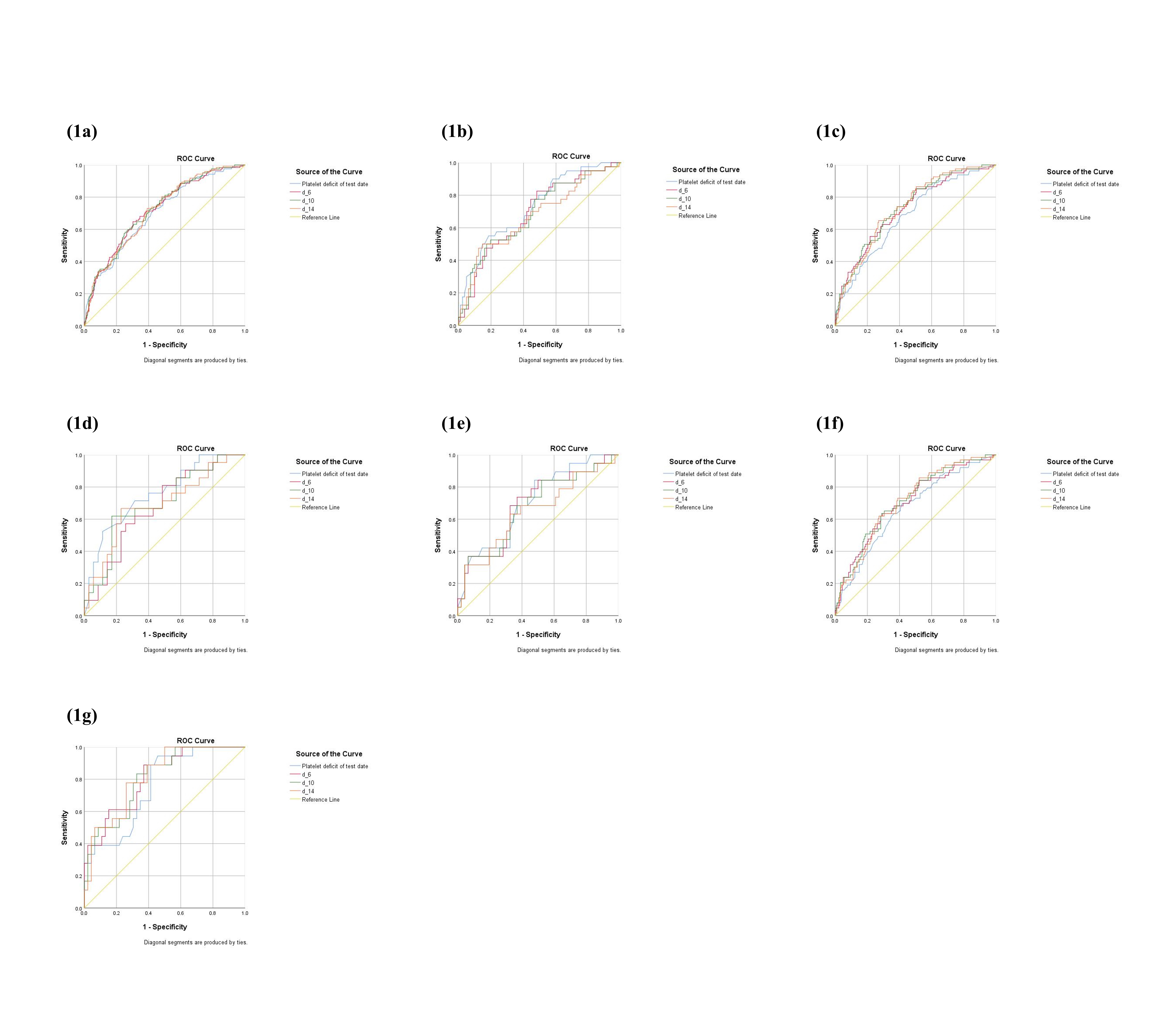

Supplement: Supplementary file 4 [file Image_2.JPEG]
